# Genetically Modified Mesenchymal Stem Cells: The Next Generation of Stem Cell-Based Therapy for TBI

**DOI:** 10.3390/ijms21114051

**Published:** 2020-06-05

**Authors:** Rami Ahmad Shahror, Chung-Che Wu, Yung-Hsiao Chiang, Kai-Yun Chen

**Affiliations:** 1 Department of Anesthesiology and Center for Shock, Trauma and Anesthesiology Research (STAR), University of Maryland, School of Medicine, Baltimore, MD 21201, USA; rami.shahror@yahoo.com; 2 Ph.D. Program for Neural Regenerative Medicine, College of Medical Science and Technology, Taipei Medical University and National Health Research Institutes, Taipei 110, Taiwan; 3 Center for Neurotrauma and Neuroregeneration, Taipei Medical University, Taipei 110, Taiwan; johnwu@tmu.edu.tw; 4 TMU Neuroscience Research Center, Taipei Medical University, Taipei 110, Taiwan; 5 Department of Neurosurgery, Taipei Medical University Hospital, Taipei 110, Taiwan; 6 Department of Surgery, School of Medicine, College of Medicine, Taipei Medical University, Taipei 110, Taiwan

**Keywords:** mesenchymal stem cells, traumatic brain injury, cell therapy, gene therapy, genetic modification, neurogenesis

## Abstract

Mesenchymal stem cells (MSCs) are emerging as an attractive approach for restorative medicine in central nervous system (CNS) diseases and injuries, such as traumatic brain injury (TBI), due to their relatively easy derivation and therapeutic effect following transplantation. However, the long-term survival of the grafted cells and therapeutic efficacy need improvement. Here, we review the recent application of MSCs in TBI treatment in preclinical models. We discuss the genetic modification approaches designed to enhance the therapeutic potency of MSCs for TBI treatment by improving their survival after transplantation, enhancing their homing abilities and overexpressing neuroprotective and neuroregenerative factors. We highlight the latest preclinical studies that have used genetically modified MSCs for TBI treatment. The recent developments in MSCs’ biology and potential TBI therapeutic targets may sufficiently improve the genetic modification strategies for MSCs, potentially bringing effective MSC-based therapies for TBI treatment in humans.

## 1. Introduction

Traumatic brain injury (TBI) is the most common form of head injury and is estimated to result in death or hospital admission for more than 10 million people annually worldwide [1,2]. The leading causes of TBI are transportation-related incidents, falls, and violence [3,4]. Although the incidence of TBI is independent of age and gender, the highest TBI incidence was in males aged 20–30 years [4]. The direct and indirect expenses of TBI in the United States in 2000 alone were estimated to be over $76 billion, highlighting the financial burden of TBI for health care systems and individuals [5]. Monotarget therapy for TBI was not effective due to the multifactorial and heterogeneous nature of TBI since various manifestations occur in different parts and timepoints post-injury [6]. Therefore, an ideal therapeutic strategy would have a multitarget, simultaneous action to induce a robust treatment for TBI [6]. One promising therapeutic option that has multitarget, simultaneous action is mesenchymal stem cell (MSC)-based therapy due to their secretion of neurotrophic factor and other neuroprotective factors [7].

MSCs have gained significant attention as an emerging therapeutic intervention for various CNS diseases and injuries such as spinal cord injury, multiple sclerosis, ischemic stroke, as well as TBI [8,9,10,11,12,13,14,15,16]. MSCs are considered promising therapeutic cells for clinical utility, owing to their ease of isolation, immunosuppression features that allow allogeneic transplantation without immunosuppression, and lack of ethical controversies [17]. However, the therapeutic potency of MSCs in vivo is affected by their poor survival, homing, and the functionality of the cells at the injured tissue. Advances in MSCs’ biology, molecular biology, and genetic engineering have opened up new approaches to improve MSC-based therapy potency. Genetic modification of MSCs to enhance their survival, homing, and sustainable release of therapeutic factors is particularly attractive.

In this review, we will highlight the pathology of TBI and the recent application of MSCs for TBI treatment. We will also discuss the approaches for the genetic modification of MSCs. The latest application of genetically modified MSCs for TBI treatment will also be addressed.

## 2. Search Strategy and Selection Criteria

The databases used to select the most relevant papers included in this article were: Google Scholar, Web of Science, MEDLINE, and PubMed. Keywords for searching (selection criteria): mesenchymal stem cells, traumatic brain injury, cell therapy, genetic modification, neurogenesis. We set dates of searching: 1996–2019. We selected only the available English articles for performing this study.

## 3. Neuroinflammatory Cascade of TBI

TBI is a heterogeneous and complex brain injury that occurs due to the occurrence of external mechanical force. The external mechanical force can transfer to the head directly (collision, assault) or indirectly (sudden acceleration-deceleration of the head). TBI results in two main injuries, based on the cellular and histological pathology: (1) primary injury that occurs when an external mechanical force transferred to the head, and (2) secondary injury cascades that are activated by the primary injury. The primary injury of TBI occurs at the moment of insult and results in rapid necrotic cell death. However, the secondary injury of TBI is more destructive, characterized by a progressive apoptotic cell death that becomes evident within several hours to days after trauma and can extend for weeks to months after the initial injury [18].

**Figure 1:**
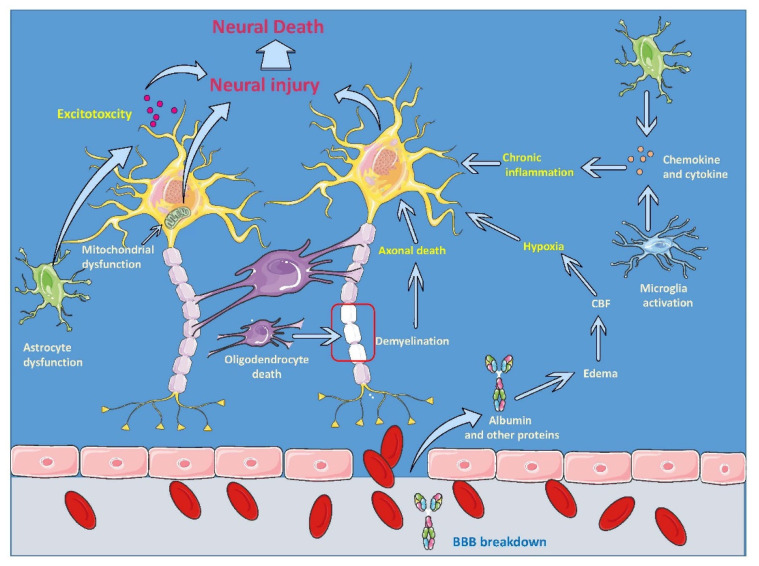
Traumatic brain injury (TBI) pathobiology. The primary insult of TBI results in blood-brain barrier (BBB) breakdown and necrotic death of neurons. Following the BBB breakdown, perfusion, and increased edema, leading to increased hypoxia results in neural injury and death. Aberrant neurotransmitter release from the injured neurons leads to excitotoxic cell injury and death. Astroglial and microglial cell activation releases numerous cytokines and chemokines, both leading to chronic inflammation. The further cellular injury occurs due to oligodendrocyte death and axonal death. Abbreviations: BBB, blood-brain barrier; CBF, cerebral blood flow; TBI, traumatic brain injury.

Advances in the diagnosing of CNS’s pathological conditions and innovative pharmacological protocols helped to discover the molecular cascade and expand our understanding of the pathological basis of neurological diseases and brain injuries [19]. Extensive research has elucidated the associated molecular cascades that underpin the neuronal dysfunction and death evident in the secondary injury of TBI; these include glutamate excitotoxicity, ischemia, intracellular calcium dysregulation, oxidative stress, and neuroinflammation (see Figure 1) [20,21,22,23,24,25,26,27,28]. TBI can result in loss of other brain cells such as astrocytes, which can affect these cells’ functions and viability [29,30]. There is increasing recognition that TBI heightens the risk of several neurodegenerative diseases [31,32].

Inflammation is a hallmark of the secondary injury following TBI that contributes to neuronal damage and affects neural repair mechanisms. Inflammation is considered as the major cause of secondary cell death following TBI [33,34,35]. Following the initial injury, injured axons produce debris that triggers an excessive and continuous systemic inflammatory response that leads to immediate cell death [18,36,37,38]. The injured cells release both pro-inflammatory cytokines which activate the microglia, the main resident immune cells in the brain. Previously, one study demonstrated that activated microglial cells can be detected at eight weeks in chronic TBI and is associated with CA3 cell loss, and dysfunctional cell proliferation in the hippocampus [33]. The compromised BBB and cytokines release allows the infiltration and activation of peripheral immune cells such as leukocytes, and macrophages that can transform into microglia that add a further immune response to TBI [39,40,41,42,43]. Although the microglia can eliminate cell debris and promote tissue remodeling, the increased inflammatory responses can lead to increased white matter injury and cell death due to the excessive secretion of pro-inflammatory cytokines that trigger the secondary injury cascade that can last for years post-injury [44,45,46,47,48,49,50,51,52,53]. Ramlackhansingh and colleagues showed that chronic inflammation following the initial impact of TBI might persist for up to 17 years post-TBI [54]. These observations was confirmed in humans by postmortem histological evidence that showed that microglial activation could be present after many years following TBI [47].

## 4. MSC-Based Therapy for TBI

### 4.1. MSCs’ Biology

MSCs are multipotent stromal cells that can differentiate into a few unique mesenchymal cell types. The International Society for Cellular Therapy proposed the following criteria to define human MSCs: (1) plastic adherent in vitro; (2) positive for the surface markers CD105, CD73, and CD90 and negative for CD45, CD34, CD14 or CD11b, CD79a or CD19, and HLA-DR; and (3) capable of differentiating into osteoblasts, adipocytes, and chondroblasts in vitro under standard differentiating conditions [55].

MSCs are most often isolated from bone marrow by a density gradient centrifugation method. Other sources of MSCs are umbilical cord blood (UCB), adipose tissue or placenta, and dental pulp. However, MSCs have heterogenic phenotypes due to the different source tissue microenvironments, and this may confer distinct functional properties on the cells [56]. For instance, adipose tissue-derived MSCs displayed a pronounced expression of the surface markers CD34+, PODXL, CD36, CD49f, CD106 and CD146, and more adipogenic differentiation capability compared to bone marrow-derived MSCs [57]. Another study examined the immunoregulatory properties of placenta-derived MSCs and other cells derived from the same source and showed that placenta-derived MSCs were more immunosuppressive [58]. Differences in cell donors might affect cells’ characterization. For example, one study showed that the expression of interleukin-1α in MSCs isolated from young rats was eight-fold higher than in cells from aged rats [59].

MSCs are known to have paracrine and autocrine activities for injured tissues in the brain due to their multifunctional secretome [60]. Few studies have demonstrated that MSCS were able to differentiate into neuronal cells following transplantation in brain tissue [61,62,63]. However, the neurological benefits observed in these studies attributed to MSCs’ paracrine and cytokine actions rather their differentiation into neuronal cells due to low engraftment of MSCs into brain tissue [63,64]. Thus, maintaining MSCs’ stemness for prolonged period post-transplantation that allows the cells to release paracrine effectors and trophic factors for more extended periods is essential to improve the functional outcome in the injured brain. Although there is no clinical trial has reported the development of cancer from experimentally given MSCs, potential tumorigenicity, promoting tumor growth and metastasis have reported in in vitro and preclinical model [65,66,67]. However, its essential to consider the the immunological status of experimental animals in studies designed for evaluation of tumorigenicity of MSC as many studies use immune-deficient animal models.

The mechanism of action of MSCs depends on their homing ability toward the injured tissues and the secretion of trophic factors that facilitate the endogenous repair processes. The most well-known secreted factors by MSCs are the brain-derived neurotrophic factor (BDNF), basic fibroblast growth factor (bFGF), glial cell-line derived neurotrophic factor (GDNF), nerve growth factor (NGF), and vascular endothelial growth factor (VEGF) [68]. The factors released by MSCs were found to mitigate local inflammation, reduce free radical levels, inhibit apoptosis, and promote the angiogenesis, proliferation, and differentiation of the injured tissue’s stem cells [69].

### 4.2. MSCs’ Application for TBI Treatment

**Figure 2:**
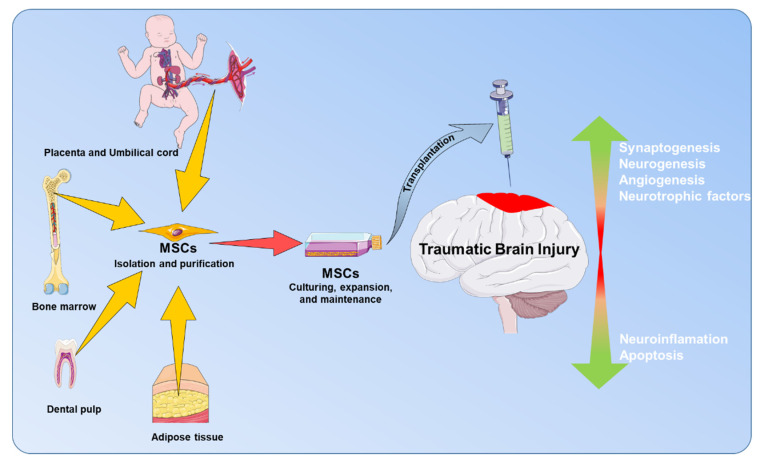
Summary of the therapeutic effects of mesenchymal stem cells (MSCs) in TBI. Prior to transplantation, MSCs can be isolated easily from different sources such as adipose tissue, placenta and umbilical cord, bone marrow, or dental pulp. Transplantation of MSCs can increase synaptogenesis, neurogenesis, angiogenesis, and neurotrophic factors at the injured brain tissue after TBI insults. Furthermore, MSCs can inhibit neuroinflammation, and apoptosis and thereby promote neuroprotective or neurorestorative effects, as well as improve functional outcomes after TBI.

MSCs are emerging as a potential stem cell-based therapy for TBI (see Figure 2) [62,70,71,72,73,74]. Direct transplantation of MSCs into the injured brain tissue during cranial repair operations in TBI patients has shown no adverse effects, indicating the safe profile of MSCs in clinical application for the treatment of TBI [75]. Direct delivery of MSCs to injured tissue in the brain, or indirect delivery through intravenous or intra-arterial injections, can cause significant amelioration of TBI-induced motor and cognitive deficits in preclinical models. Accumulated preclinical studies demonstrated that systemically infused MSCs were able to bypass the blood-brain barrier and elevate the expression of neuroprotective factors in the brain after TBI [70]. Besides, MSC transplantation following TBI has shown that these cells can migrate and survive in the injury site, where they contribute to neuroprotection, neural repair, and motor function [72,76]. Furthermore, MSCs’ secretome can modulate the inflammatory response following TBI by decreasing cytokines’ expression in the brain tissue [77].

MSCs can reduce neuroinflammation and improve functional recovery after TBI [78]. Intravenous MSC transplantation two hours after TBI in a weight-drop rat TBI model reduced the peripheral infiltration of neutrophils (MPO+) and CD3+ lymphocytes, activation/infiltration of macrophages/microglia, as well as pro-inflammatory cytokines [78]. These anti-inflammatory effects of MSCs were associated with reduced apoptosis in the injured tissues and early functional improvement. MSC transplantation found to improve functional outcomes in TBI via inhibiting the microglial polarization toward the M1 pro-inflammatory microglial phenotype [79]. MSCs-based therapy was able to augment the excessive acute pro-inflammatory response to the level needed for debris clearance. Such regulation of the pro-inflammatory response can prevent the development of chronic neuroinflammation and promote neuroprotection in TBI. The anti-inflammatory effects of MSCs are potential targets for further enhancement of MSCs’ therapeutic effects in TBI by reducing secondary injury, which ultimately leads to functional improvement.

Decreased hippocampal neurogenesis following TBI at the acute phase is well-established, demonstrated by a robust reduction in immature neurons in the hippocampus [80,81,82]. A recent study reported that TBI impaired hippocampal neurogenesis at the chronic phase, evidenced by a significant decrease in immature neurons in the hippocampus, which correlated with hippocampal-dependent learning and memory deficits [83]. Several studies showed that TBI also impaired hippocampal neurogenesis in terms of dendrite development arborization, morphology, and functional integration into the hippocampal neuronal network [83,84]. Several studies have demonstrated that TBI can lead to axonal sprouting in hippocampal mossy fiber pathways that have been linked with abnormal excitation and post-traumatic seizures [85,86,87,88,89]. In human, TBI insult can cause abnormal axonal growth that leads to aberrant hippocampal mossy fiber sprouting [90].

Furthermore, the impairments in dendrite development, arborization, and morphology of immature neurons in the hippocampus ultimately lead to post-traumatic impairment in learning and memory by affecting the functional synaptic integration and disrupting signaling transduction, which is essential for action potential propagation in neurons [91]. MSCs can promote and enhance the endogenous regeneration of the injured tissue. For instance, Munoz and colleagues showed that MSCs facilitate the proliferation, migration, and differentiation of endogenous neural stem cells (NSCs) following direct engraftment of MSCs in the hippocampus [92]. Several studies demonstrated that showed that MSC transplantation following TBI rescued the impairment in the dendrite length of the newborn neuron in the injured hippocampus [83]. Besides, the secretome from MSCs promoted the survival and proliferation of endogenous neural stem cells in the injured brains of mice with TBI [62].

Intravenous MSC delivery for TBI therapy has been increasingly used in preclinical studies. Although the majority of these studies suggest that significant numbers of administered MSC are initially trapped in the lungs, MSC reaching the damaged brain after crossing the BBB improved neurobehavioral scores were still reported [70,93,94]. Several MSCs tracking studies showed that intravenously administered MSC tend to migrate from the lungs to other tissues such as the spleen and liver, or sites of injury in short periods [95,96,97,98,99]. Several studies have indicated that MSCs might cross the BBB via molecular mechanisms that involve adhesion molecules, chemokines, and proteases using in vitro BBB models [100,101,102,103,104]. For example, Steingen et al. showed that MSCs could integrate into the endothelium via the adhesion molecules VCAM-1/VLA-4 and β1 integrin then cross the endothelial of BBB into host tissue through the use of plasmic podia [100]. In TBI, MSC administration increased the production of Tissue Inhibitor of Matrix Metalloproteinase 3 (TIMP3) that attenuate the increased BBB’s permeability after injury in an animal model [105]. Although These studies suggest that MSCs can cross the BBB to the site of injury or inflammation, the compromised the integrity BBB flowing TBI insult might result in a passive accumulation MSC in the brain via entrapment [106].

## 5. Genetically Modified MSC-Based Therapy for TBI

As pointed out in the previous section, MSC-based therapy for TBI is a promising option to facilitate recovery of the injured tissue in the brain. However, due to the harsh microenvironment of the injured tissue and the complexity of TBI injury, the development of new strategies to improve the homing and survival and paracrine properties of MSCs at the injury site is urgently needed. Genetic modification is a promising strategy to maximize MSCs’ therapeutic capacity in vivo. Genetic modification of MSCs is usually achieved using viral vectors, although the use of non-viral vectors is on the rise.

### 5.1. Viral Vector-Mediated Genetic Modification

Viral vectors are the most common and efficient vectors for the genetic modification of host cells due to their natural ability to infect the cells, bypass the cellular barriers and deliver genetic material into the host cell’s nucleus. Integrative viral vectors, such as retrovirus and lentivirus, are very efficient vectors for the genetic modification of host cells as they can deliver and integrate the gene of interest into the host cell’s genome. While retrovirus can only transduce dividing cells with a smaller loading capacity (8 kb), lentivirus can transduce dividing and non-dividing cells with larger loading (9 kb) [107,108]. Although integrative vectors are very efficient in genetic modification, they raise safety concerns due to the possibilities of insertional mutagenesis induction and proto-oncogene activation in the host cells [109]. Non-integrative viral vectors are vectors that can infect the host cells without integration with the host genome. Adeno-associated virus (AAV) is the most common non-integrative viral vector used for genetic modification due to its non-pathogenic features, strong expression of the gene of interest, and low risk of insertional mutagenesis. Although viral vector-mediated genetic modification may be appealing for MSC-based therapy, the long-term safety of viral gene therapy remains a concern. Furthermore, the transduced cells might present viral antigens that could potentially activate an immune response in vivo following transplantation [110]. A benefit-to-risk ratio should be considered before such cell therapy be practical.

### 5.2. Non-Viral Vector-Mediated Genetic Modification

Advancements in molecular biology and genetic engineering have led to the synthesis and design of non-viral vectors that can deliver large genetic materials into the cells. Other advantages of non-viral vectors are the possibility to control the expression of the gene of interest via regulatory manipulation, their reduced immunotoxicity, and is easier and cheaper to produce on a large scale compared to viral vectors. Preconditioning can improve the intrinsic therapeutic properties of MSCs against the harsh microenvironment within its transplanted milieu, which is predominant in TBI injured tissue. Several studies have reported that preconditioning can enhance the interaction between MSCs and the innate/adaptive immune responses. Chen et al. demonstrated that culturing MSCs under hypoxic conditions improves the cells’ expression of more antiapoptotic proteins, IL-8 and IL-6 [111]. Similar results were reported by Jiang et al. that showed hypoxic conditions improved IL-10 and FasL in vitro in MSCs [112]. The enhanced immunomodulation, in turn, mitigate the inflammation and encourage the injured tissue repair and regeneration.

Non-viral integrative vectors such as excisable systems and transposons often disrupt the host genome, leading to limited applications for these vectors in the genetic modification of therapeutic cells. Non-integrative vectors such as episomal vectors are less toxic compared to integrative viral vectors [113]. Although the non-viral vector-based methods for genetic modification offer an exciting promise, their use in the genetic modification of therapeutic cells might be hampered by transient expression of the gene of interest and vector damage following cell infection [114]. Although non-viral genetic modification methods have low transfection efficiency compared to viral-based methods, its ability to alleviate the safety concerns with reduced immunogenicity makes it more likely to enter the clinical trials.

### 5.3. Application of Genetically Modified MSCs for TBI Treatment

Genetic modification of MSCs involves inducing the overexpression of factors that are critical for MSCs’ therapeutic effects [115]. Such factors can be proteins that enhance the homing and survival of MSCs and the secretion of trophic factors that facilitate the restorative processes at the injury site. Overexpression of factors that have anti-inflammatory and neuroprotective effects is a particularly promising modification for TBI therapy. Genetic modification of MSCs will have more advantages compared to classical gene therapy strategies, which are based on direct infection of the targeted tissue in vivo by lentiviral or AAV vectors. In contrast, MSCs are considered a safer approach for gene therapy. Also, the viral transfection of MSCs can be easily controlled ex vivo and limited to one to two viral vectors per MSC genome to follow the FDA regulation for stem cells and gene-based therapy trials. Importantly, genetic modification can custom MSCs for the treatment of a wide range of brain injuries such as stroke and TBI.

**Figure 3:**
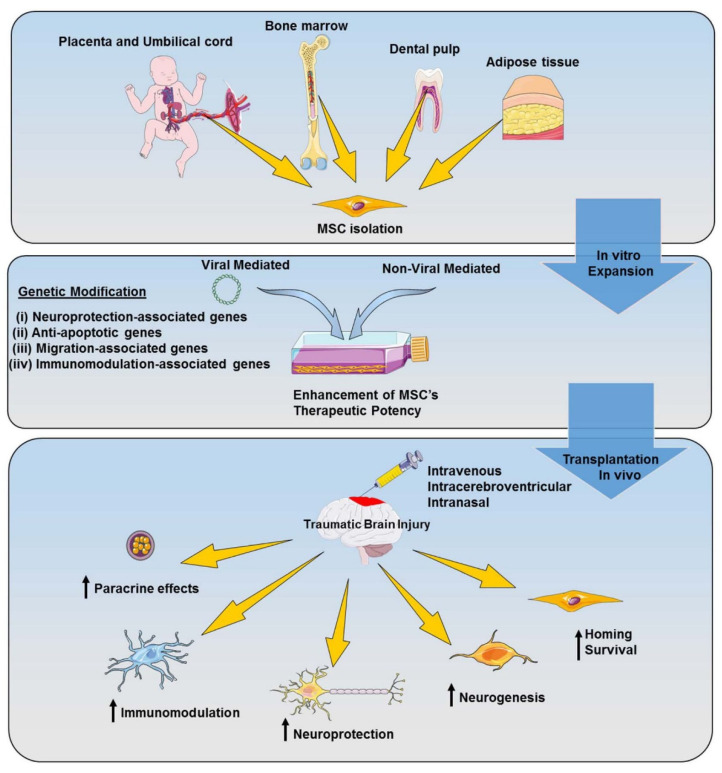
Improving MSCs’ therapeutic potential for TBI via genetic modification. Illustration of possible MSC sources in humans and the possible targets for genetic modification in vitro. Following transplantation, the genetically modified MSCs are able to improve the homing, survival, and paracrine effects of MSCs, enhance neurogenesis, and enable neuroprotection and immunomodulation at the injury site in TBI.

There is growing evidence supporting the efficiency of using genetically modified MSC-based therapy for TBI in preclinical models (Figure 3). One promising approach for using genetically modified MSCs for TBI treatment is to overexpress factors that have anti-inflammatory effects, as inflammation is one of the most well-established mechanisms of the secondary injury of TBI. The application of genetically modified MSCs to overexpress an anti-inflammatory cytokine, in particular, IL-10, to reduce inflammation has been evaluated in preclinical models of various injuries and disorders, including arthritis, autoimmune encephalomyelitis, ischemia-reperfusion injury in the lung, graft-versus-host disease, and ischemic stroke [116,117,118,119,120]. A recent study showed that using genetically modified MSCs to overexpress IL-10 mitigated TBI deficits by reducing inflammation, preventing apoptosis and tissue loss, and reducing the production of TNF-αin a rat model of TBI [121]. The increase of IL-10 in accordance with the decrease in TNF-α promotes a shift in the macrophages/microglia activation state, from classical to alternative CD163-activated cells.

Another promising strategy for enhancing the therapeutic potential of MSCs by genetic modification is by enhancing their homing abilities to the injured tissue. MSCs can migrate to sites of TBI injury [72,76]. A recent study showed that transplanted MSCs can be detected at the injury site as early as 24 h later and can survive for 28 days post-injury in a mouse model of TBI [83,122]. Recent studies have shown that genetic modification of MSCs is a feasible approach to improve MSCs’ homing abilities in TBI. For instance, MSCs expressing CXCR4 are highly attracted to the potent chemoattractant stromal cell-derived factor-1 (SDF-1), which was found to be dramatically overexpressed at the zone of lesions by astrocytes and endothelial cells. The SDF-1/CXCR4 axis promotes MSCs homing to injury sites in the brain and other organs.Wang and colleagues showed that genetically modified MSCs that overexpress CXC chemokine receptor 4 (CXCR4) enhanced homing abilities to the injury site in a TBI model Due to the improved homing of these cells, paracrine secretion of cytokines and growth factors was improved, leading to enhanced vasculogenesis and neuroprotection, improved blood supply, recovery of axon connectivity, and behavioral ability in treated mice. Another promising target to improve MSCs homing is fibroblast growth factor 21 (FGF21), a metabolic regulator that exhibits neuroprotective effects and promotes cells’ migration in vitro. Previous studies have shown that FGF21 improves cell migration in various cells type in vitro, including fibroblasts and cardiomyocytes. For instance, treatment with FGF21 mimics compound was able to improve human umbilical vein endothelial cells (HUVECs) migration. This study showed that FGF21 treatment-induced migration via the activation of eNOS/PI3K/AKT pathways. Another study showed that FGF21 promotes cells’ migration via the β-catenin signaling cascade and c-Jun N-terminal kinase (JNK) signaling, an essential regulator of cell migration, in fibroblasts in vitro [123,124,125]. A recent study showed that FGF21 overexpression in MSCs by genetic engineering enhanced the MSCs migration in vitro and improved the homing abilities of MSCs to the injury site in a mouse model of TBI [122]. However, the exact mechanism by which FGF21 improved the homing of MSCs is still unclear.

Even if the implanted cells are successfully migrated to the site of injury in the brain, their neuroprotective function within the harsh microenvironment is found to be reduced. Overexpressing neuroprotective genes such as BDNF, glucagon-like peptide-1 (GLP-1), FGF21 has had the dual benefit of promoting the homing and survival of transplanted MSCs as well as the paracrine factor-induced recovery and neuroprotection of the host’s brain-injured area. In one study, MSCs isolated from the umbilical cord and genetically modified to overexpress BDNF increased their ability to migrate to and survive in cerebral tissues, and mitigated neurological deficits more efficiently than MSCs alone in rats with TBI [126]. Cerebral transplantation of encapsulated MSCs that overexpress glucagon-like peptide-1 (GLP-1), a neuroprotective substance against excitotoxicity in vitro and in vivo that exhibits antioxidant effects, were able to reduce the neuronal cell loss in the hippocampus and cortical neuronal and glial defects in TBI rat models [127]. These effects were less pronounced in animals treated with MSCs alone. Recently, a study showed that the transplantation of MSCs that overexpress FGF21 improved the cognitive functions at the chronic phase following TBI in a mouse model [83]. Although the treatment with MSCs alone also showed partial functional recovery, treatment with MSC-FGF21 exhibited more pronounced functional recovery. MSC-FGF21 treatment was associated with enhanced hippocampal neurogenesis, enhanced FGF21 protein levels, and reduced morphological deficits in immature neurons in the injured hippocampus.

Accumulated studies have identified promising therapeutic cellular targets for TBI, especially those who play a critical role in the pathogenesis of traumatic brain injury. In vitro and preclinical studies that explored these targets showed promising therapeutic results that able to ameliorates TBI-induced cellular and functional consequences. For example, exogenous neuroprotective factors like CuZn-SOD and other neurotrophins such as NGF, BDNF, and FGF-2 exhibited neuroprotective effects in vitro, and in vivo [128,129,130,131,132,133]. Their rapid deactivation, off-target effects on the PNS, and low permeability through the BBB would limit their application in clinical use for TBI [134]. However, that makes them excellent candidates for genetically modified MSC-based therapy that would improve MSCs’ potency and therapeutic potential. Other examples of potential candidates for MSC genetic modification are miRNAs such as miR-195, miR-24, and miR-19b due to their role in the neuronal apoptosis, regeneration, and neurodegenerative processes. Several reports showed significant alterations of miRNAs expression hippocampus and plasma following TBI [135,136]. MSCs offer an excellent delivery system for miRNAs through their exosomes that can bypass BBB, and maintain the stability and functionality of the miRNA due to their lipid bilayers. These targets and factors are promising future candidates to develop target-specific genetically modified MSC-based therapy and enhance MSCs’ potency to mitigate TBI consequences.

The risk profile of genetically modified MSCs-based therapy for TBI depends on many risk factors, which include the type of genetic modification, the target of the inserted gene, their differentiation potency, and proliferation capacity, the homing ability of MSCs, the long-term survival of engrafted cells, the route of administration. Currently, there is no clinical experience with genetically modified MSCs-based therapy for TBI. Based on MSCs’ of immunomodulation characteristics and viral vector-mediated genotoxicity, the risks associated with tumorigenesis are considered high. In contrast, the vast majority of small-sized clinical trials conducted with MSC in TBI, or direct injection of viral vector into the human brain has not reported significant health concerns [75,137,138,139,140,141,142,143]. These clinical evidences suggest that use genetically modified MSC, via viral or non-viral-based modification methods, could be relatively safe for clinical application.

## 6. Conclusions

Numerous studies have outlined the beneficial effects of genetically modified MSC-based therapy for TBI. Genetic modification of MSCs enhances their viability and proliferative, migratory, and functional properties, thus increasing their therapeutic potential. Importantly, genetic modification of MSCs offers a safe, targeted, and robust option for gene delivery in gene-based therapy. The findings, as mentioned earlier, reveal that genetically modified MSC-based therapy could lead to the translation of preclinical studies into effective and safe targeted therapies for TBI.
